# Cohort study of long‐term survival and quality of life following pelvic exenteration

**DOI:** 10.1002/bjs5.75

**Published:** 2018-05-22

**Authors:** D. Steffens, M. J. Solomon, J. M. Young, C. Koh, R. L. Venchiarutti, P. Lee, K. Austin

**Affiliations:** ^1^ Surgical Outcomes Research Centre Royal Prince Alfred Hospital Sydney New South Wales Australia; ^2^ Institute of Academic Surgery Royal Prince Alfred Hospital Sydney New South Wales Australia; ^3^ Sydney Medical School University of Sydney Sydney New South Wales Australia

## Abstract

**Background:**

Pelvic exenteration (PE) is the preferred treatment available for selected patients diagnosed with locally advanced or recurrent cancer confined to the pelvis. Currently, the majority of the literature reports only on short‐term survival and quality‐of‐life (QoL) outcomes. The aim of this prospective cohort study was to describe long‐term survival and QoL outcomes following PE.

**Methods:**

This was a cohort study of consecutive patients undergoing PE from 1994 to 2016 at a major teaching hospital in Sydney, Australia. From 2008, consenting patients were also included in a prospective QoL study. Main outcomes were long‐term survival and QoL assessed with SF‐36^®^ and FACT‐C questionnaires. Survival was estimated using the Kaplan–Meier method.

**Results:**

Some 515 patients underwent PE for locally advanced or recurrent cancer. The cumulative 5‐ and 10‐year overall survival rates were 48·6 and 37·8 per cent respectively. The survival estimates were significantly higher for patients with advanced primary rectal cancer (P = 0·045) and those in whom a clear resection margin was achieved (P < 0·001). Some 287 patients were enrolled into the QoL study. Response rates at baseline, 6 months and 5 years were 92·0, 70·0 and 33 per cent respectively. Patients had recovered to their preoperative QoL status by 6 months and, among survivors, QoL remained essentially unchanged during the 5‐year follow‐up.

**Conclusion:**

Patients who underwent PE owing to advanced primary rectal cancer or achieved a clear resection margin had a greater chance of survival. Overall, QoL returned to baseline within 6 months after surgery.

## Introduction

For patients with locally advanced or recurrent cancer confined to the pelvis, including rectal, gynaecological origin and urological cancer, complete resection with clear margins (R0 resection) provides the best chance of survival^1^. Pelvic exenteration (PE) was first described in 1948 as a palliative procedure for advanced and recurrent pelvic malignancy. PE is a radical but morbid procedure that may involve the complete or partial resection of pelvic viscera, vessels, muscles, nerves and parts of the bony pelvis^2^. Advances in imaging, surgical techniques and postoperative management have moved PE from a palliative to a curative procedure in appropriately selected patients^3^. This is, however, at the expense of high complication rates and prolonged recovery after surgery^4^ ^5^.

Despite the magnitude of the operation, previous studies have demonstrated an acceptable short‐term overall survival rate and reasonable quality‐of‐life (QoL) outcomes^6^. An overall 5‐year survival rate of between 30 and 60 per cent has been reported, with QoL outcomes returning to preoperative levels in the first 6–9 months after surgery^7^ ^8^. Little is known about long‐term patient survival and QoL outcomes^9^. Information on long‐term outcomes will be useful for patient counselling and decision‐making regarding whether or not to proceed with this radical surgery. The aim of this study was to describe long‐term overall survival and QoL outcomes after PE.

## Methods

This was a cohort study of consecutive patients who underwent PE at Royal Prince Alfred Hospital, a major public hospital in Sydney, Australia. All patients diagnosed with advanced primary or recurrent pelvic cancer who had partial or complete PE between 1 September 1994 and 31 December 2016 were included in the study. For patients who had more than one PE surgery, only data relating to the first operation were included in this study. Patients aged less than 18 years at surgery, or who underwent PE for conditions other than cancer (including chronic sepsis or benign pathology), were excluded from analysis.

Clinical data on all patients who underwent PE during the study interval were collected by a research officer using a standard data collection form. Collected data included details of the primary tumour, operative and admission details, histopathology findings and postoperative complications. A partial PE was defined as *en bloc* resection of up to three anatomical components of the pelvis, and a complete PE as *en bloc* resection of all four pelvic anatomical components. Death was verified either by the hospital medical records or by consultation within the Registry of Births Deaths and Marriages (http://www.bdm.nsw.gov.au/). Survival was defined as time from the PE surgery to date of death by any cause or last contact, and censored at August 2017. Ethical approval was obtained from the Sydney Local Health District Human Research Ethics Committee (SLHD HREC) (approval number X13‐0283), and informed consent was waived. For the prospective QoL study commencing in 2008, all patients with locally advanced or recurrent pelvic cancer undergoing PE surgery were invited to participate. Patients presenting with inadequate English‐language skills to complete outcome measures or cognitive impairment such that they were unable to provide informed consent were excluded from the study. Informed consent was obtained from all patients participating in the prospective QoL study (SLHD HREC approval number X16‐0272).

### Quality of life

Consenting eligible patients completed the Short Form 36 (SF‐36^®^; Quality Metric, Lincoln, Rhode Island, USA)^10^ and the Functional Assessment of Cancer Therapy – Colorectal (FACT‐C)^11^ instruments at baseline (before surgery) and at 6, 12, 18, 24, 30, 36, 48 and 60 months after PE surgery. Both instruments are widely used and well validated in the assessment of QoL, and have been used previously in colorectal and PE populations. The SF‐36^®^ instrument consists of eight QoL domains that can be integrated in two summary scales: the physical component score (PCS) and the mental component score (MCS). These are norm‐based scores with an average of 50 for the population and a standard deviation of 10. Higher scores indicate better QoL. The FACT‐C instrument provides one total score (possible range 0–136), and consists of five domains (physical, social, emotional and functional well‐being, and an additional domain addressing colorectal cancer‐specific concerns including gastrointestinal symptoms and questions relating to ostomy appliances). For both the SF‐36^®^ and FACT‐C instruments, a higher score represents better QoL. Patient responses to the QoL questionnaires were scored according to algorithms provided in the user manuals for the instruments^12^ ^13^. QoL trajectories were also reported according to the type of PE (complete *versus* partial). Follow‐up QoL data were collected to August 2017.

### Outcomes

The main outcomes were long‐term overall survival and QoL after PE. In addition, the influence of cancer presentation (advanced primary *versus* recurrent rectal cancer), resection margin (R0 *versus* R1/R2), pelvic exenteration (complete *versus* partial), participation in QoL study (yes *versus* no) and year of exenteration (1994–2011 *versus* 2012–2016) on survival was investigated. The QoL trajectories were also reported according to the type of pelvic exenteration (complete *versus* partial).

### Statistical analysis

Categorical data are presented as frequencies (percentage), and continuous data as mean(s.d.) values for normally distributed data and as median (range) for skewed data. Overall survival was estimated using the Kaplan–Meier product limit method^14^, with median survival estimates presented alongside 95 per cent confidence intervals. Differences in survival estimates between groups were assessed using the log rank test, and patients who were alive at last contact were censored. Normally distributed data were compared between groups using Student's *t* test, skewed data were compared with the Mann–Whitney *U* test, and dichotomous data with the χ^2^ test. All *P* values were two‐sided, and statistical significance was taken at *P* < 0·050. Mean scores from the SF‐36^®^ (PCS and MCS) and FACT‐C (total score) are presented for each time point with 95 per cent confidence intervals. All statistical calculations were conducted using SPSS^®^ version 22 (IBM, Armonk, New York, USA).

## Results

**Figure 1:**
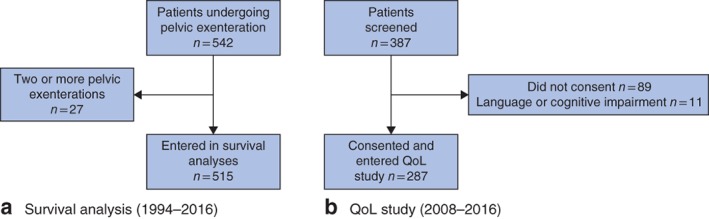
Flow diagram showing the numbers of **a** patients who had pelvic exenteration surgery between 1994 and 2016, and **b** the subgroup of patients who had exenteration in 2008–2016 and were enrolled in the quality‐of‐life (QoL) study

Between September 1994 and December 2016, 542 patients underwent PE surgery for locally advanced or recurrent pelvic cancer. Twenty‐seven patients had repeated PE operations owing to recurrence. Thus, clinical and survival data relating to 515 patients were analysed (*Fig*. 1 *a*). Of the 387 patients undergoing surgery between 2008 and 2016, 287 (74·2 per cent) consented and were enrolled into the QoL study (*Fig*. 1 *b*). Of these patients, 267 (93·0 per cent) completed the baseline study questionnaire before surgery.

The mean(s.d.) age of all patients at surgery was 59·6(12·6) years, and most patients were men (53·4 per cent). Some 26·8 per cent presented with advanced primary rectal cancer, and 35·1 per cent with recurrent rectal cancer. Complete PE was performed in 40·4 per cent of patients, and an R0 resection margin was achieved in 68·5 per cent. Sepsis was the most common complication, affecting 40·6 per cent of patients. Median duration of hospital stay was 21 (range 2–196) days. Patients enrolled into the QoL study were more likely to be men and to have a complete exenteration, a longer hospital stay, and higher rates of in‐hospital postoperative complications. Characteristics of the overall sample and those of the subgroup that participated in the QoL study are presented in *Table* 1.

**Table 1:** Demographic and clinical characteristics of patients who underwent pelvic exenteration

	Overall (*n* = 515)	Patients in QoL study (*n* = 287)	Patients not in QoL study (*n* = 228)	*P* ‡
Age (years)*	59·6(12·6)	59·5(12·5)	59·8(12·8)	0·813§
Sex ratio (M : F)	275 : 240	176 : 111	99 : 129	< 0·001
Presentation				0·002
Advanced primary rectal	138 (26·8)	77 (26·8)	61 (26·8)	
Recurrent colorectal	181 (35·1)	119 (41·5)	62 (27·2)	
Advanced primary, other	85 (16·5)	41 (14·3)	44 (19·3)	
Recurrent, other	111 (21·6)	50 (17·4)	61 (26·8)	
Neoadjuvant therapy				0·059
Radiotherapy only	26 (5·0)	15 (5·2)	11 (4·8)	
Chemotherapy only	37 (7·2)	23 (8·0)	14 (6·1)	
Chemoradiotherapy	205 (39·8)	128 (44·6)	77 (33·8)	
None	200 (38·8)	99 (34·5)	101 (44·3)	
Resection margin				0·432
R0	353 (68·5)	201 (70·0)	152 (66·7)	
R1/R2	141 (27·4)	77 (26·8)	64 (28·1)	
Extent of exenteration				< 0·001
Complete	208 (40·4)	145 (50·5)	63 (27·6)	
Partial	296 (57·5)	139 (48·4)	157 (68·9)	
Blood loss (ml)†	2000 (0–25 000)	2500 (0–25 000)	1200 (0–24 000)	< 0·001¶
Duration of hospital stay (days)†	21 (2–196)	22 (5–175)	20 (2–196)	0·037¶
Postoperative complications				
Sepsis	209 (40·6)	121 (42·2)	88 (38·6)	0·413
Wound	114 (22·1)	68 (23·7)	46 (20·2)	0·339
Cardiovascular	113 (21·9)	67 (23·3)	46 (20·2)	0·388
Gastrointestinal	104 (20·2)	67 (23·3)	37 (16·2)	0·046
Urological	104 (20·2)	62 (21·6)	42 (18·4)	0·372
Ostomy	99 (19·2)	64 (22·3)	35 (15·4)	0·047
Respiratory	93 (18·1)	61 (21·3)	32 (14·0)	0·034
Neurological	71 (13·8)	45 (15·7)	26 (11·4)	0·162
Depression	28 (5·4)	15 (5·2)	13 (5·7)	0·813
Haemorrhage	16 (3·1)	7 (2·4)	9 (3·9)	0·327
Other	269 (52·2)	151 (52·6)	118 (51·8)	0·846
Complication rate (in hospital) (%)	418 (81·2)	246 (85·7)	171 (75·0)	0·003
1‐year all‐cause mortality	63 (12·2)	35 (12·2)	28 (12·3)	0·977

Values in parentheses are percentages unless indicated otherwise; values are

* mean(s.d.) and †median (range). Percentages may not total 100 per cent owing to missing data. QoL, quality of life.

‡ χ^2^ test, except

§ Student's *t* test and

¶ Mann–Whitney *U* test (patients enrolled in QoL study *versus* those not enrolled).

### Survival

**Figure 2:**
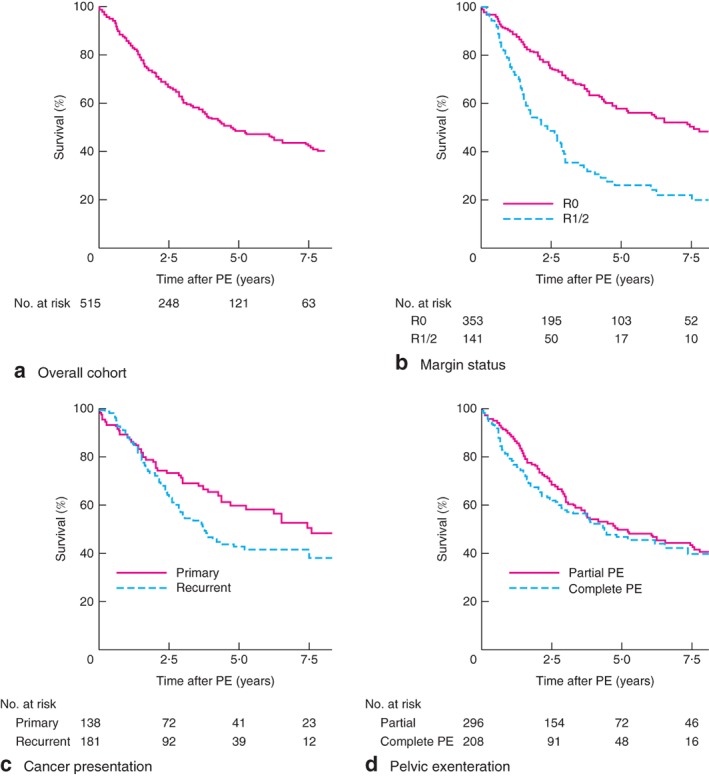
Kaplan–Meier estimates of long‐term survival in patients undergoing pelvic exenteration (PE) surgery: **a** overall cohort, **b** according to margin status, **c** according to cancer presentation (primary or recurrent), **d** according to partial or complete PE. **b** P < 0·001, **c** P = 0·045, **d** P = 0·128 (log rank test)

The median length of follow‐up was 2·4 (range 0·7–21·6) years. Of the 515 patients, 63 died within 12 months after the surgery. The 1‐year all‐cause mortality rate was therefore 12·2 per cent. Kaplan–Meier survival curves are presented in *Fig*. 2 and *Fig. S1* (supporting information). Median overall survival was 4·7 (95 per cent c.i. 3·5 to 6·0) years, and 5‐ and 10‐year survival rates were 48·6 and 37·8 per cent respectively (*Fig*. 2 *a*). Patients with an R0 resection margin had better median survival than those with R1/R2 surgical margins (7·6 (5·3 to 10·0) and 2·4 (1·6 to 3·2) years respectively; *P* < 0·001) (*Fig*. 2 *b*). Patients with advanced primary rectal cancer demonstrated better long‐term survival outcomes in comparison with patients who presented with recurrent rectal cancer: median overall survival 7·6 (3·5 to 11·8) *versus* 3·8 (2·9 to 4·7) years respectively (*P* = 0·045) (*Fig*. 2 *c*).

No secular trends in overall survival were observed between patients having surgery between 1994 and 2011 and those operated on in the last 5 years (2012–2016) (median survival 4·8 and 4·4 years respectively). Differences in survival were not significant, with 5‐year survival rates for these groups of 48·9 and 39·3 per cent respectively (*P* = 0·913) (*Fig. S1*, supporting information).

### Quality of life

Among surviving patients, response rates to the QoL questionnaires ranged from 92·0 per cent (264 of 287) at baseline (before surgery) to 33 per cent (28 of 85) at 5 years (*Fig. S2*, supporting information). Reasons for non‐response to the QoL study are depicted in *Fig. S2*, accounting for patients who died during the study, those who had not reached a time point of data collection, and missing data for unknown reasons.

**Figure 3:**
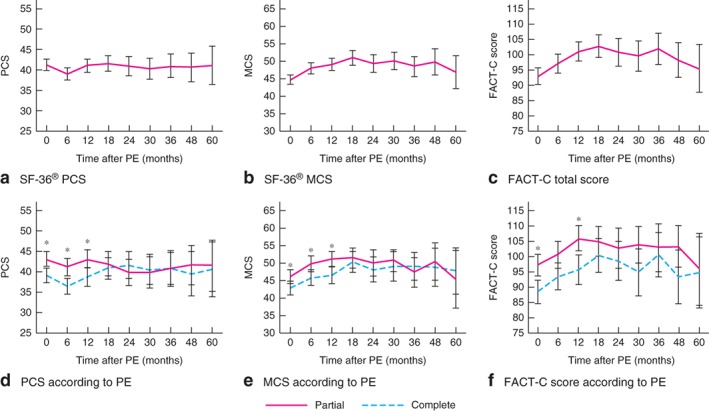
Quality‐of‐life scores over 5 years in patients who underwent pelvic exenteration (PE): **a** SF‐36^®^ physical component score (PCS), **b** SF‐36^®^ mental component score (MCS), **c** Functional Assessment of Cancer Therapy ‐ Colorectal (FACT‐C) total score, **d** PCS according to type of PE (partial or complete), **e** MCS according to type of PE, **f** FACT‐C total score according to type of PE. Mean values are shown with 95 per cent confidence intervals. SF‐36^®^ scores range from 0 to 100, FACT‐C scores from 0 to 136; higher scores represent better quality of life. *P < 0·050 (Student's t test)

The mean PCS from the SF‐36^®^ essentially remained stable over the 5‐year follow‐up (*Fig*. 3 *a* and *Table* 2), from 41·2 (95 per cent c.i. 39·9 to 42·5) at baseline to 41·1 (36·4 to 45·5) 5 years after surgery. The PCS reached its lowest point at 6 months after operation (mean PCS 38·9, 37·4 to 40·4), but had recovered to 41·1 (39·5 to 42·7) by 12 months, remaining stable from that time point. The MCS from the SF‐36^®^ increased slightly from 44·6 (43·2 to 46·0) at baseline to 50·9 (48·9 to 53·0) at 18 months, remaining essentially unchanged thereafter (*Fig*. 3 *b* and *Table* 2).

**Table 2:** Quality‐of‐life scores over 5 years

	SF‐36^®^ PCS*		SF‐36^®^ MCS*		FACT‐C total score†	
	*n*	Mean score	*n*	Mean score	*n*	Mean score
Baseline	262	41·2 (39·9, 42·5)	262	44·6 (43·2, 46·0)	264	92·8 (90·1, 95·5)
6 months	183	38·9 (37·4, 40·4)	184	47·9 (46·3, 49·5)	183	97·2 (94·0, 100·3)
12 months	154	41·1 (39·5, 42·7)	154	49·0 (47·3, 50·7)	153	101·0 (97·8, 104·2)
18 months	111	41·5 (39·5, 43·4)	110	50·9 (48·9, 53·0)	118	102·8 (99·2, 106·4)
24 months	85	40·9 (38·6, 43·2)	85	49·3 (46·8, 51·8)	89	100·7 (96·2, 105·3)
30 months	76	40·3 (37·8, 40·3)	76	50·0 (47·4, 52·6)	78	99·5 (94·6, 104·4)
36 months	60	41·0 (38·1, 43·8)	60	48·4 (45·6, 51·3)	58	101·9 (96·7, 107·0)
48 months	42	40·6 (37·1, 44·2)	41	49·8 (46·1, 53·5)	42	98·1 (92·4, 103·8)
60 months	27	41·1 (36·4, 45·5)	27	46·8 (42·0, 51·6)	28	95·4 (87·7, 103·1)

Values in parentheses are 95 per cent confidence intervals.

* Possible range for SF‐36^®^ physical component scale (PCS) and mental component scale (MCS): norm‐based scores with an average of 50 for the population and a standard deviation of 10;

† possible range for FACT‐C: 0–136; a higher score represents better quality of life.

Similar to the MCS, the mean total score of the FACT‐C instrument increased from 92·8 (95 per cent c.i. 90·1 to 95·5) at baseline to 102·8 (99·2 to 106·4) at 18 months. The total score remained essentially unchanged until a slight increase to 101·9 (96·7 to 107·0) at 36 months, and progressively decreased to 95·4 (87·7 to 103·1) at 5 years (*Fig*. 3 *c* and *Table* 2).

At baseline and at 6 and 12 months after surgery, patients who had a partial exenteration had significantly higher PCS and MCS values from the SF‐36^®^ (*Fig*. 3 *d,e*). Patients who underwent partial PE had significantly higher FACT‐C scores at baseline and 12 months after discharge (*Fig*. 3 *f*).

## Discussion

Patients requiring PE surgery invariably have advanced and often aggressive cancer, with significant effects on QoL. In this study 5‐ and 10‐year overall survival estimates of 48·6 and 37·8 per cent respectively were observed. Achieving clear (R0) resection margins confers a significant survival advantage. Patients presenting with advanced primary rectal cancer had better 5‐ and 10‐year overall survival rates (60·1 and 46·1 per cent respectively) compared with patients with recurrent rectal cancer (rates of 42·9 and 33·9 per cent respectively). QoL was restored to preoperative status within the first 6 months and remained essentially unchanged for up to 5 years after surgery.

The 5‐year overall survival rate for patients undergoing PE who presented with locally advanced pelvic malignancy has been reported as between 30 and 60 per cent, although most of the literature is based on small retrospective studies^1^ ^15^, ^16^. Over the study period (1994–2016), survival outcomes remained stable for patients undergoing PE surgery. This finding may be explained by the evolving case mix of patients seen at the authors' institution since the inception of the PE service, which grew from performing fewer than ten PE surgeries per year in 1994–2003 to consistently performing over 50 exenterations yearly since 2014. Although the case mix of patients over this period was not investigated in this study, it is reasonable to assume that, as surgical experience increases, more advanced and technically challenging cases are referred and accepted for surgery; this may explain the stable survival outcomes observed over time.

Trends in PCS values over 5 years reflect the physically morbid nature of a radical procedure such as PE surgery, and the impact of long‐term sequelae of treatment that patients may experience. PE is associated with high morbidity, reflected in high rates of postoperative complications. In the present study, 81·2 per cent of all patients, and 85·7 per cent of those enrolled in the prospective QoL study, experienced at least one postoperative complication. A complication rate of 81 per cent among 148 PE patients enrolled in a QoL study was reported previously^7^ ^17^. Most studies^18^, ^19^, ^20^, ^21^ report complication rates between 49 and 75 per cent, although some have reported complication rates as low as 26 per cent^8^ and as high as 94 per cent^22^. These findings should be taken into consideration by both surgeons and patients during patient selection and counselling for a procedure such as PE surgery, to prepare patients and carers emotionally for life after the operation.

This was a single‐centre study, which limits the generalizability to other centres, and thus the findings may need to be viewed with caution. Although the SF‐36^®^ and FACT‐C instruments are valid and reliable measures of QoL, no specific instrument currently incorporates all issues that affect patients undergoing PE surgery^9^. A PE‐specific instrument may better reflect the QoL of patients undergoing this procedure. Missing data for the self‐reported QoL assessment may have introduced bias to the results, particularly for those patients who had surgery before commencement of the QoL study in 2008, and the specific exclusion from the QoL study of patients unable to speak English may not reflect specific issues faced by migrant populations. In addition, loss to follow‐up (including that due to death) over the study period resulted in a small proportion of patients completing the QoL questionnaires at 5 years compared with baseline, reducing the accuracy of the estimates, as reflected in the increasingly widening confidence intervals over time. The potential influence of missing QoL data on the findings of this study is open to conjecture. Patients may have experienced recurrent or distant disease, preventing them from completing the questionnaires, which may have resulted in an overestimation of QoL, particularly at later time points with fewer participants. Additionally, the burden of completing multiple questionnaires regularly, particularly for older patients, was a common reason for withdrawal from the study. Missing QoL data were not imputed for analysis of long‐term QoL outcomes, and therefore the results presented in this study represent the experience of surviving patients who continued to respond to questionnaires. The aim of this study was not to compare the QoL experience between patient groups, who may have a different survival experience. This was considered a more valid approach than imputing missing data, particularly with respect to the large amount of data missing owing to deaths and participants not reaching time points. Complete exenteration was associated with poorer physical and mental QoL in the first year after surgery. As significantly more patients enrolled in the QoL study had a complete PE compared with patients who were not enrolled, the QoL scores may in fact be lower than would have been the case if all patients had been enrolled in the study.

Further investigation into surgical trends for this procedure and the influence on patient‐centred outcomes is thus warranted. Exploring the specific experiences of patients undergoing PE surgery using a tailored QoL tool is necessary, as these patients usually have at least one postoperative complication and report reduced physical and mental QoL for long periods after surgery.

## Supporting information


**Fig. S1** Long‐term survival estimates for patients undergoing pelvic exenteration surgery
**Fig. S2** Number of patients followed up during the quality‐of‐life study (n = 287)
