# Supplementary material for: Cohort study of long‐term survival and quality of life following pelvic exenteration
Source: BJS Open. 2018 May 22;2(5):328–35. doi: 10.1002/bjs5.75 (PMC6156168; doi:10.1002/bjs5.75)
Supplement: Supplementary file 1 — Fig. S1 Long‐term survival estimates for patients undergoing pelvic exenteration surgery Fig. S2 Number of patients followed up during the quality‐of‐life study (n = 287) [file BJS5-2-328-s001.docx]

**BJS5_75**

**A cohort study of long-term survival and quality of life following pelvic exenteration**

**D. Steffens, M. J. Solomon, J. M. Young, C. Koh, R. L. Venchiarutti, P. Lee and K. Austin**


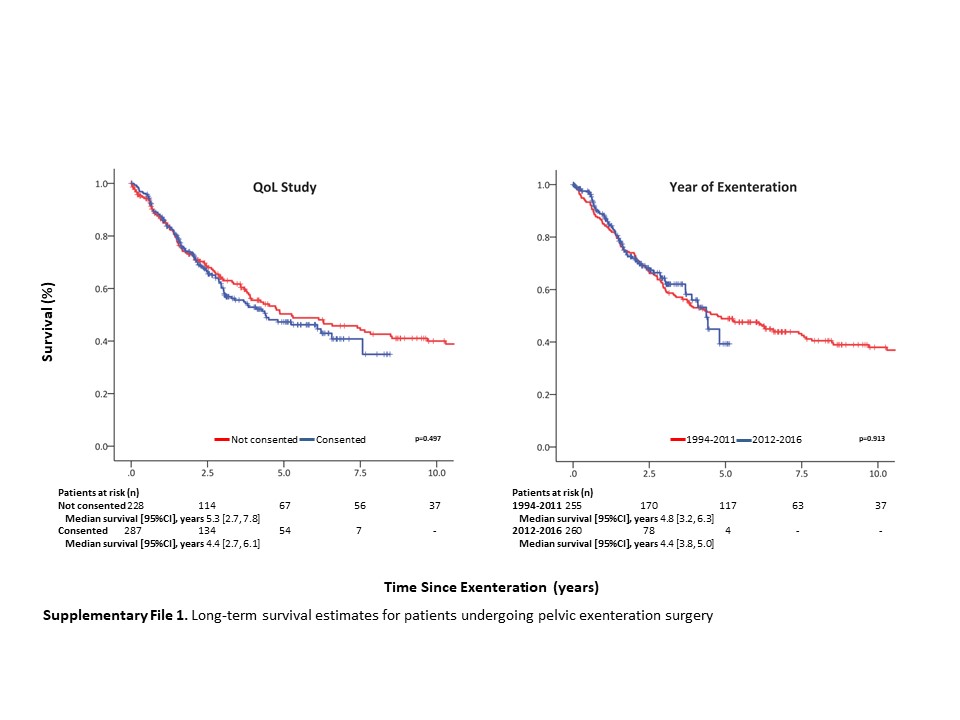
**Fig. S1** Long-term survival estimates for patients undergoing pelvic exenteration surgery


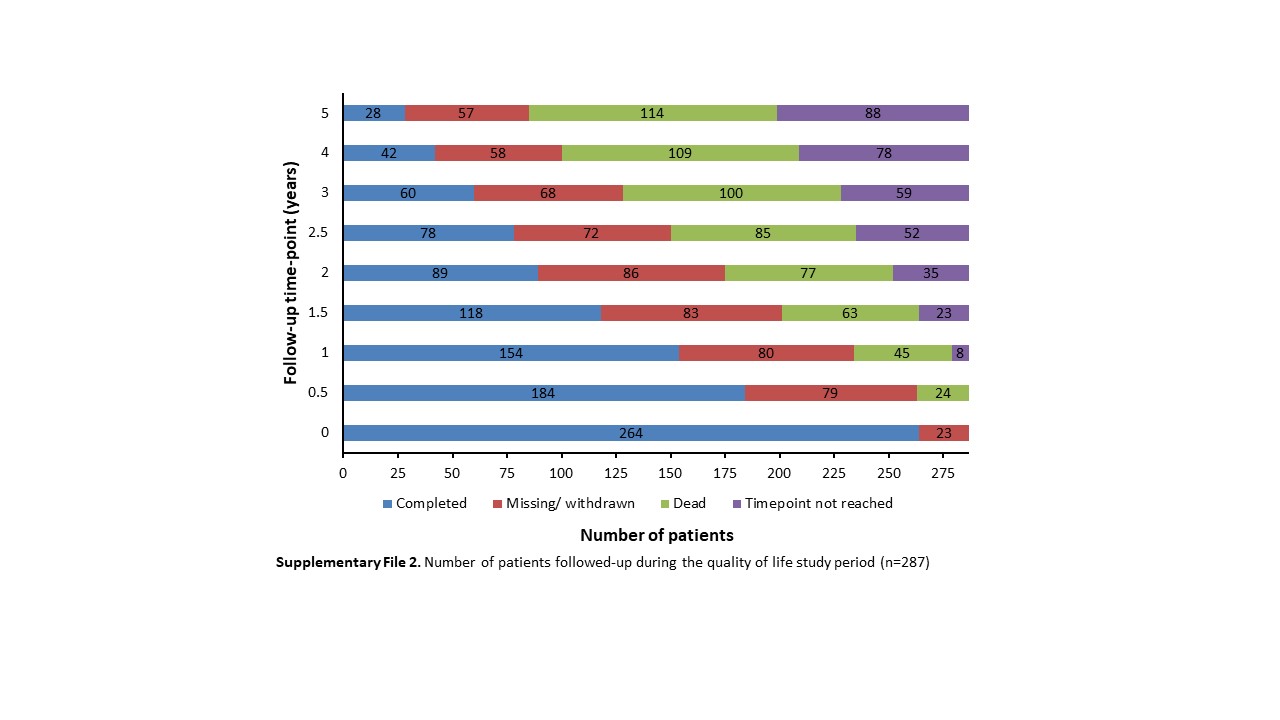


**Fig. S2** Number of patients followed up during the quality-of-life study (*n* = 287)
